# Genomics Sheds Light on Metabolism of Cryptic Marine Microbes

**DOI:** 10.1371/journal.pbio.0040123

**Published:** 2006-03-21

**Authors:** Liza Gross

In 1977 Carl Woese and George Fox expanded our appreciation of microbial diversity by analyzing the genetic sequence of a molecule (ribosomal RNA) found in all cells. They discovered that species previously classified as bacteria, called methanogenic bacteria, possessed unique enzymes and an unusual metabolism based on reducing carbon dioxide to methane. These traits were foreign to both “uber” domains of life, Eurkaryota and Bacteria, prompting Woese to create a new category, which he called Archaebacteria ( *archae* means ancient in Greek), acknowledging a metabolism that would have suited the putative conditions on earth over 3 billion years ago.

Archaeal groups have been found in a wide array of habitats—from boiling sulfur pits, salt marshes, and hydrothermal vents to frosty Antarctic surface waters, mud flats, and freshwater habitats—yet less than 0.1% of expected species have been characterized.

In a new study, genetic analysis offers clues to the fundamentals of archaeal life and some insight into how these organisms can exist in such diverse environments. Steven Hallam, Edward DeLong, and their colleagues enlist genomics techniques to identify the pathways used by the marine sponge symbiont *Cenarchaeum symbiosum* to accomplish life’s most essential processes: energy metabolism and carbon assimilation. And by comparing the *C. symbiosum* genome sequence with sequences extracted from environmental samples collected from diverse ocean habitats, they show that planktonic Crenarchaeota share many of the same genetic components.

Many archaeal species can use inorganic compounds (rather than sunlight, like plants) as an energy source for carbon synthesis, earning them the unwieldy name of chemolithoautotroph. Several lines of evidence suggest that planktonic Crenarchaeota, significant components of the marine ecosystem, assimilate carbon in this way and that they might use ammonia (NH3) as an energy source, since they inhabit ammonia-rich Antarctic waters and are associated with high nitrite concentrations. (Nitrite is a by-product of ammonia oxidation.)

To search for genetic clues to carbon and energy metabolism in Crenarchaeota, the researchers extracted *C. symbiosum* DNA from its host sponge and constructed a DNA library for sequencing the symbiont’s genome. Hallam et al. then searched for representative genes linked to pathways associated with autotrophic carbon assimilation. They found many components of two pathways: the 3-hydroxypropionate cycle and the reductive tricarboxylic acid (citric acid) pathway (TCA). Both cycles involve a multistep series of chemical reactions that convert inorganic compounds—in this case, carbon dioxide—into organic carbon molecules. Though some components of the 3-hydroxypropionate cycle were missing in *C. symbiosum*, enough elements (including core proteins) were found to support a modified version of this pathway for carbon assimilation, using carbon dioxide.

In eukaryotes, the TCA cycle links the oxidative breakdown of carbon compounds with biosynthesis and energy metabolism. In prokaryotes, the process is reversed, with the oxidation of inorganic compounds (such as carbon dioxide) providing the means for carbon assimilation. Again, though some TCA components were missing, Hallam et al. found evidence suggesting that *C. symbiosum* could use partial TCA reactions to produce biosynthetic precursors. It’s possible that other genes take the place of the missing components or that the TCA and 3-hydroxypropionate pathways overlap.

The researchers next searched for genes that might play a role in generating energy from ammonia oxidation (also called nitrification because ammonia is converted to nitrite). The *C. symbiosum* genome contains many genes associated with nitrification in bacteria, including genes that encode the subunits of ammonia monooxygenase, which catalyzes the first step in converting ammonia to nitrite. The researchers could not find evidence for several proteins that function downstream in this pathway, however, suggesting that the symbiont uses alternative mechanisms to effect nitrification. Supporting this possibility, the researchers found candidate genes that might take the place of some of these missing elements, as well as others that could protect the cell from the toxic nitrites generated by ammonia oxidation.

How did the genes identified here compare with planktonic Crenarchaeota gene sequences? To find out, Hallam et al. searched GenBank (the National Institutes of Health genetic sequence database) and an environmental database containing gene sequences collected from the Sargasso Sea and other ocean waters for similar sequences. Many components of both the 3-hydroxypropionate and the TCA cycle were found in the environmental database. And each of the *C. symbiosum* genes studied here were most closely related to sequences from planktonic Crenarchaeota—suggesting that even though these archaeal lineages evolved under different selective pressures, they rely on similar metabolic strategies.

Overall, Hallam et al. argue that, though the forms show significant divergence at the nucleotide level, *C. symbiosum* and planktonic Crenarchaeota share “striking” similarities in the identity and organization of their genes. And with gene sequences linked to fundamental processes like carbon assimilation and energy metabolism in *C. symbiosum*, researchers can probe parallel processes in marine Crenarchaeota—an endeavor that will likely reveal the vital role these once-enigmatic organisms play in the carbon and nitrogen cycles of marine ecosystems.

## 

**Figure:**
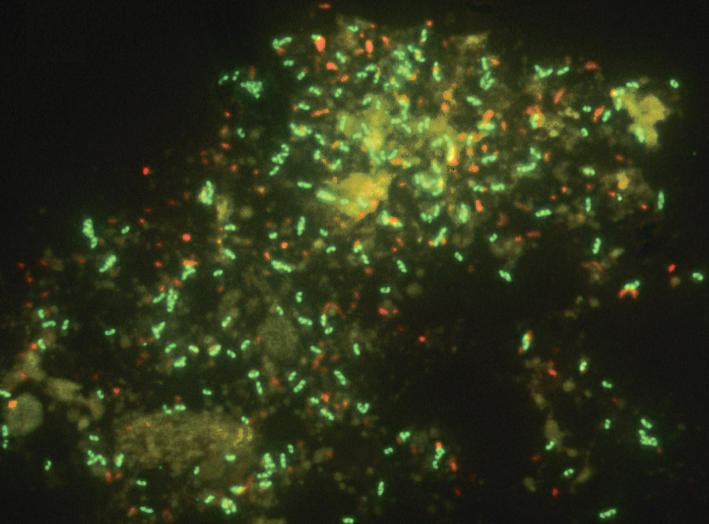
The bright green cells represent the archaeal sponge symbiont, C. symbiosum, stained with a fluorescently labeled C. symbiosum-specific DNA probe. The red cells represent surrounding bacteria also found in the sponge host tissue.

